# Modeling the Behavioral Response of Dentists to COVID-19 and Assessing the Perceived Impacts of Pandemic on Operative Dentistry Practices in Pakistan

**DOI:** 10.3389/fpubh.2022.904838

**Published:** 2022-06-13

**Authors:** Syeda Afshan Manzoor, Abdul-Hakeem Alomari

**Affiliations:** ^1^Department of Operative Dentistry & Endodontics, Bakhtawar Amin Medical & Dental College, Multan, Pakistan; ^2^Biomedical Engineering Department, College of Engineering, Imam Abdulrahman Bin Faisal University (IAU), Dammam, Saudi Arabia

**Keywords:** COVID-19, COM-B model, dentistry, operative dentistry, Pakistan

## Abstract

COVID-19 pandemic has affected dentistry in unprecedented ways. This study investigates the perceived effects of the pandemic on operative dentistry procedures and dentistry profession in Pakistan and the factors that determine the behavioral changes among dentists to adapt to the “new normal.” A Capability Opportunity Motivation-Behavioral model (COM-B) was utilized to investigate the factors that determine the behavior of dentists in Punjab, Pakistan to adhere to COVID-19 standard operating procedures (SOPs). Using social media, an online questionnaire was sent to operative dentistry professionals in Pakistan, and 312 responses were received. 81.4% of the respondents believed that the COVID-19 pandemic has severely affected the level of care provided to the patients, 66% were extremely worried about the risk of contagion during clinical practices, and more than 75% of the respondents opined that the pandemic has led to an increased emphasis on disinfection and oral hygiene instructions. The multiple regression model suggests that the behavior of Pakistani dentists to adhere to the COVID-19 SOPs is significantly affected by their Capabilities (β = 0.358) and Opportunities (β = 0.494). The study concluded that dentists in Punjab, Pakistan are concerned about the risk of contagion and report a serious concern about consequences such as financial loss and inappropriate care of patients. The current study results can feed the policymaking in Pakistan and other developing countries. Facilities and training to improve dentists' opportunities and capabilities can improve their ability to cope with the COVID-19 challenges.

## Introduction

Worldwide, healthcare professionals are on the first lines in fighting the COVID-19 pandemic. They have become highly vulnerable to COVID-19 transmission, constituting 9% of all the infected cases (1, 2). Dental practitioners are among high-risk healthcare professionals because of direct exposure to blood and saliva (3). The Occupational Safety and Health Administration (OSHA) devised an occupational risk pyramid which shows the vulnerability of healthcare professionals based on their exposure to COVID-19 virus (https://www.osha.gov/Publications/OSHA3990.pdf; United States, Department of Labor). According to the OSHA (Department of Labor, USA), dentists are at “very high risk.” Earlier studies confirmed that aerosol transmission and respiratory droplets are potential pathways of COVID-19 transmission (4).

Dental professionals are especially vulnerable when carrying out aerosol-generating procedures (AGPs) on infectious patients (5). Evidence suggests that while a dentist is treating a patient on a dental chair, the highest levels of aerosol contaminants are within 50–60 cm of the face of the patient. Furthermore, aerosols are highest on face masks of patients, and around their nose and eyes (6). The aerosol contaminants generated by ultrasonic devices can remain in the air for half an hour after the procedure (7). Thus, dentists are highly vulnerable to infection because of close proximity to the patient, contact with patients' blood and saliva as well as due to the use of instruments that generate aerosols (8, 9).

Evidence suggests that returning to work after the COVID-19 outbreak requires dentists and healthcare professionals to adopt a behavioral change to adhere to COVID-19 SOPs during clinical practices (5). The main SOPs include the use of personal protective equipment (PPE) in line with government advice, use of robust infection prevention and control procedures and use of high-power rubber suction and rubber dam where an aerosol generating procedure is necessary (5, 8, 9). This is especially true for operative dentistry professionals since several operative dentistry procedures involve AGPs, which makes dentists critically exposed to infection risk (5, 10, 11). Furthermore, COVID-19 also led to increased anxiety and practice modification as well as had economic consequences for dentists (12). Thus, it is critical to identify and understand the factors driving behavioral change in operative dentistry professionals.

Capability Opportunity Motivation-Behavior model (COM-B) has been used to study and understand the behavioral change in dentistry (5). The COM-B consists of three components that drive behavioral change: Capability, Opportunity, and Motivation (13, 14). In the proposed COM-B model, Capability is defined as the internal factors that enable an individual to engage in a given behavior. Opportunity is defined as the external variables or factors that allow an individual to engage in a given behavior. Motivation consists of a “conscious motivation” (intentional plans to engage in a given behavior) and “automatic motivation” (defined as an individual's habitual or instinctive response) (13). In another study (4), COM-B is utilized as a framework to understand the factors underpinning behavioral change for intervention.

Due to a high exposure to infection, wearing of PPE was made mandatory for healthcare professionals all around the world. PPE includes protective eyewear, N-95 mask, full-length gowns covering body from head to toe, air-purifying respirators, and surgical gloves. Compared to the countries such as New Zealand (15), Canada (9), and Saudi Arabia (16), dentists in developing countries have shown far less knowledge and compliance to the COVID-19 infection control SOP's (10). Although there is evidence of positive attitude toward the use of PPE by dentists in some developing countries including Iraq (6), in most developing countries, attitude toward the use of PPE during COVID-19 outbreak has not been encouraging.

In developing countries including Pakistan, the usage of PPE and adherence to the COVID-19 infection control SOP's is still quite challenging (17). Limited evidence is available which suggests that one of the key factors influencing the attitude of dentists toward the use of PPE are financial constraints and poor knowledge about the use of PPE among dentists in Pakistan (17). Studies in dental hospital in Rawalpindi and Karachi suggest that as low as 20 percent of the dental students complied to COVID-19 SOPs (18). This is an alarming situation for a country like Pakistan as dentists' lack of compliance of COVID-19 SOPs could lead to increased burden on scarce resources of the country. Therefore, it is vital to investigate how the COVID-19 pandemic has affected the dentistry profession in Pakistan and what factors underpin the behavioral change in the dentists of Pakistan.

This study aims to understand the perceived impacts of the COVID-19 pandemic on the dental profession in the Punjab province of Pakistan and understand the factors that underpin the behavioral change in the dentists to adapt to the COVID-19 SOPs. Punjab is the most populous province of Pakistan, containing more than 110 million people. Also, Punjab is the most affected province of the country by COVID-19 pandemic. So far, 506,018 confirmed cases of COVID-19 have been reported in Punjab. Of these confirmed cases, 13,560 patients died (https://covid.gov.pk/stats/punjab). Dentists working in all departments are vulnerable to COVID-19 exposure. Current study focuses on dentists who specialize and work in operative dentistry departments. This study specifically concentrated on this group of dentists as these dentists are among the most affected professionals by the challenges and risks associated with treatments frequently requiring AGPs. Although this study could have involved other professionals such as periodontists and oral hygienists, limiting this study to operative dentists allowed a more comprehensive and in-depth survey of the perception of a specific department of dentistry.

The specific objectives of this study are (a) to identify the perceived effects of COVID-19 on dentistry practices in Pakistan, (b) to identify the perceived impacts of COVID-19 on operative dentistry procedures in Pakistan, and (c) to identify the drivers of behavioral change of adherence of COVID-19 SOPs among dentists of Pakistan using COM-B model.

## Methodology

A survey-based cross-sectional study was conducted to collect data for this project. A structured questionnaire was prepared through review of literature and consultation with expert biostatisticians, operative dentists, and psychologists. The questionnaire was approved by the Institutional Research Board (IRB) of Bakhtawar Amin Dental College & Hospital Multan, Pakistan (reference BADC&H No. 300/21). The questionnaire was pre-tested once with a pilot survey of 25 respondents. The demographic profile of these 25 respondents was fairly similar to the profile of the respondents in the actual survey (i.e., of the 25 respondents, 18 were female and six were male. Eighteen respondents aged between 20 and 30 years, five respondents 30–40 years old and two respondents were above 60 years age. In terms of type of workplace, 19 respondents worked in Government hospitals, three worked in private hospitals and three respondents worked in both Government and private hospitals). Informed consent of all respondents was obtained.

### Sample Selection

According to Pakistan Medical Dental Council Islamabad, total number of registered dentists in Pakistan are approximately 25,000 (https://www.pbs.gov.pk/sites/default/files//tables/rename-as-per-table-type/Registered%20Dental%20Doctor.pdf). Of these, 9,000 dentists are registered in Punjab (https://tribune.com.pk/story/1975950/pakistan-facing-acute-shortage-doctors). The sample size for this survey was determined by the total number of dentists in Punjab province of Pakistan (i.e., 9,000), using the sample size calculation formula proposed by Yamane (19). The confidence interval was set to 6% and a 95% confidence level was used. With these parameters, the sample size derived was 260.

### Questionnaire Development

An online questionnaire was created using Google Forms and cascaded to registered operative dentistry professionals in Punjab, Pakistan, through social media (WhatsApp groups of dentists). The survey started on May 20th, 2021. The online survey was kept open for 10 weeks. Four reminders were sent to the respondents, each after 2 weeks (through messages in the WhatsApp groups) to record their responses during this time.

The questionnaire for this study consisted of 32 questions which were divided into eight sections. A complete draft of the questionnaire is provided in the Table 1.

**Table 1:** List of sections and questions included in the questionnaire for this study.

**Section**	**Questions**	
**Demographic information**	Age (Years)	20–30. 30–40. 40–60. Above 60
	Type of workplace	Government. Private. Work in both Government and Private workplaces simultaneously
	Gender	Male. Female
	Nature of Job	Clinical. Teaching. Both clinical and teaching.
	Clinical experience (years)	Less than 1. 1–3. 3–5. 5–10. 10–15. More than 15.
	Dental education	BDS (Or equivalent). Post-graduation (In progress). Post-graduation (Completed)
**Impacts of COVID-19 pandemic on your profession**	To what extent has COVID-19 pandemic affected the ability of dentists to provide appropriate levels of care to the patients?	
	To what extent do you think COVID-19 pandemic is likely to cause loss of clinical skills in dentists?	
	To what extent do you think COVID-19 pandemic has financially affected dentists?	
	To what extent COVID-19 pandemic causes risk of contagion in dentists due to unavailability of appropriate PPE?	
**Impacts of COVID-19 pandemic on dental practices**	COVID-19 pandemic has positively impacted dental practice because it has allowed better spaced appointments	
	COVID-19 pandemic has positively impacted dental practice because it has led to more emphasis on disinfection procedures	
	COVID-19 pandemic has positively impacted dental practice because it has led to more emphasis on Oral Health Instructions (OHI)	
	COVID-19 pandemic has negatively impacted dental practices by causing reduced number of patients	
	COVID-19 pandemic has negatively impacted dental practices by causing reduced number of follow-up visits	
**Impacts of COVID-19 pandemic on procedures in operative dentistry**	COVID-19 pandemic has affected restorative procedures	
	COVID-19 pandemic has affected endodontic procedures	
	COVID-19 pandemic has affected aesthetic procedures (veneers, bleaching, etc)	
	COVID-19 pandemic has affected implants procedures	
**Opportunities to follow COVID-19 SOPs**	On a scale of 1–10, how regularly do you wear PPE during your clinical practices?	
	On a scale of 1–10, how often do you ensure that your patients follow SOPs during your clinical practices?	
	On a scale of 1–10, how often do you follow infection control measures (high volume suction, rubber dam isolation, etc) during clinical practice?	
	On a scale of 1–10, how confident you are that you have the required physical resources available to follow COVID-19 SOPs at your workplace?	
	On a scale of 1–10, how confident you are that your colleagues support you to follow COVID-19 SOPs at your workplace?	
	On a scale of 1–10, how confident you are that you have the required time available to follow COVID-19 SOPs at your clinic/hospital/workplace?	
**Motivation to follow COVID-19 SOPs**	I feel that it is my moral obligation to follow the COVID SOPs during practice.	
	I follow COVID SOPs automatically/unconsciously without reminding myself (has become a habit for me)	
	If I implement COVID-19 SOPs correctly and regularly, I will be a role model for my colleagues	
**Capability to follow COVID-19 SOPs**	I have enough physical strength to follow COVID SOPs during clinical practice	
	I have sufficient knowledge/information about how to follow COVID SOPs during clinical practice	
	Even if I commit an error, I feel confident to implement COVID-19 SOPs correctly again	

The first section recorded the demographic details of the respondents. The respondents were asked about their age, gender, location, years in dentistry professions, dental education/training, type of workplace and nature of their jobs.

The second section enquires about respondents' perceived impacts of COVID-19 pandemic on dentistry profession. All answers were recorded on a Likert scale of 1–5 where 1 = “Not at all” and 5 = “Extremely.”

The purpose of the third section was to explore the perceived impacts of pandemic situation on various dental procedures. The respondents were asked to record their answers on Likert scale, ranging from 1 to 5 (Completely disagree to Completely agree).

In the fourth section, the questionnaire inquired the participants about the perceived impacts of COVID-19 on various procedures in operative dentistry. Operative dentistry procedures include aesthetic (e.g., tooth whitening, veneers, crowns etc.), endodontics (e.g., root canal treatment), implant, and restorative procedures (e.g., tooth restoration.). The respondents were asked to record their responses on a Likert scale. The sections 2–4 were used to assess the perceived effects of the pandemic on dentistry practice and operative dentistry procedures.

Sections 5 to 8 in the survey was used to assess the behavior of respondents to adhere to COVID-19 SOPs. COM-B model is utilized to predict the “behavior to adhere to COVID-19 SOPs” using opportunities, motivation, and capabilities as independent variables. Behavior was measured using three items: (i) On a scale of 1–10, how regularly do you wear PPE during your clinical practices? (ii) On a scale of 1–10, how often do you ensure that your patients follow SOPs during your clinical practices? (iii) On a scale of 1–10, how often do you follow infection control measures during clinical practice? Opportunities were measured using three items: (i) On a scale of 1–10, how confident you are that you have the required physical resources available to follow COVID-19 SOPs at your workplace? (ii) On a scale of 1–10, how confident you are that your colleagues support you to follow COVID-19 SOPs at your workplace? (iii) On a scale of 1–10, how confident you are that you have the required time available to follow COVID-19 SOPs at your clinic/hospital/workplace? Motivation was measured using three items: (i) I feel that it is my moral obligation to follow the COVID SOPs during practice. (ii) I follow COVID SOPs automatically/unconsciously without reminding myself (has become a habit for me) (iii) If I implement COVID-19 SOPs correctly and regularly, I will be a role model for my colleagues. All questions of COM-B constructs were recorded on a Likert scale (1–10).

### Data Analysis

Descriptive statistics is used to report the frequency and percentages of the respondents in each category of the demographic variables. Stacked bar charts were used to report the proportions of the respondents who chose various levels of agreements to the questions asked in section 2–4.

In order to establish a relationship between COVID-19 SOPs adherence behavior and the opportunities, capabilities, and motivations of the respondents, a multiple linear regression analysis is utilized. The significance level was set at *p* ≤ 0.05.

Before running the regression model, Cronbach's alpha test was used to test the internal consistency in each of the four constructs (Behavior, Opportunities, Motivation and Capabilities). All data analysis were carried out in SPSS v. 21 and R statistical software. The results were then collected and performed using Statistical Package 21 for the Social Sciences SPSS® (IBM®, SPSS® Statistics, Armonk, NY, USA).

## Results

### Demographics

A total of 312 valid survey responses were received. There were no missing data on responses. Among the respondents, 74.36 % (*n* = 232) were female. Most of the respondents aged between 20 and 30 years (*n* = 210). The details of the demographics of the respondents are given in Table 2.

**Table 2:** Demographic profile of the respondents (*n* = 312).

	**Frequency (*n*)**	**Percentage (%)**		**Frequency (*n*)**	**Percentage (%)**
**Age (Years)**			**Type of workplace**		
20–30	210	67.31	Govt.	148	47.43
30–40	70	22.44	Private	90	28.84
40–60	22	7.05	Both Govt. & Private	74	23.71
Above 60 years	10	3.21			
**Gender**			**Nature of job**		
Male	80	25.64	Clinical	252	80.76
Female	232	74.36	Teaching	12	3.20
			Both Clinical & Teaching	48	15.38
**Clinical experience (years)**			**Dental education/training**		
Less than a1	26	8.33	BDS (or equivalent)	138	44.23
1–3	106	33.97	Post-graduation (in-progress)	22	7.05
3–5	106	33.97	Post-graduation (completed)	152	48.71
5–10	54	17.31			
10–15	10	3.21			
More than 15	10	3.21			

### Perceived Impacts of COVID-19 Pandemic on Your Profession

**Figure 1:**
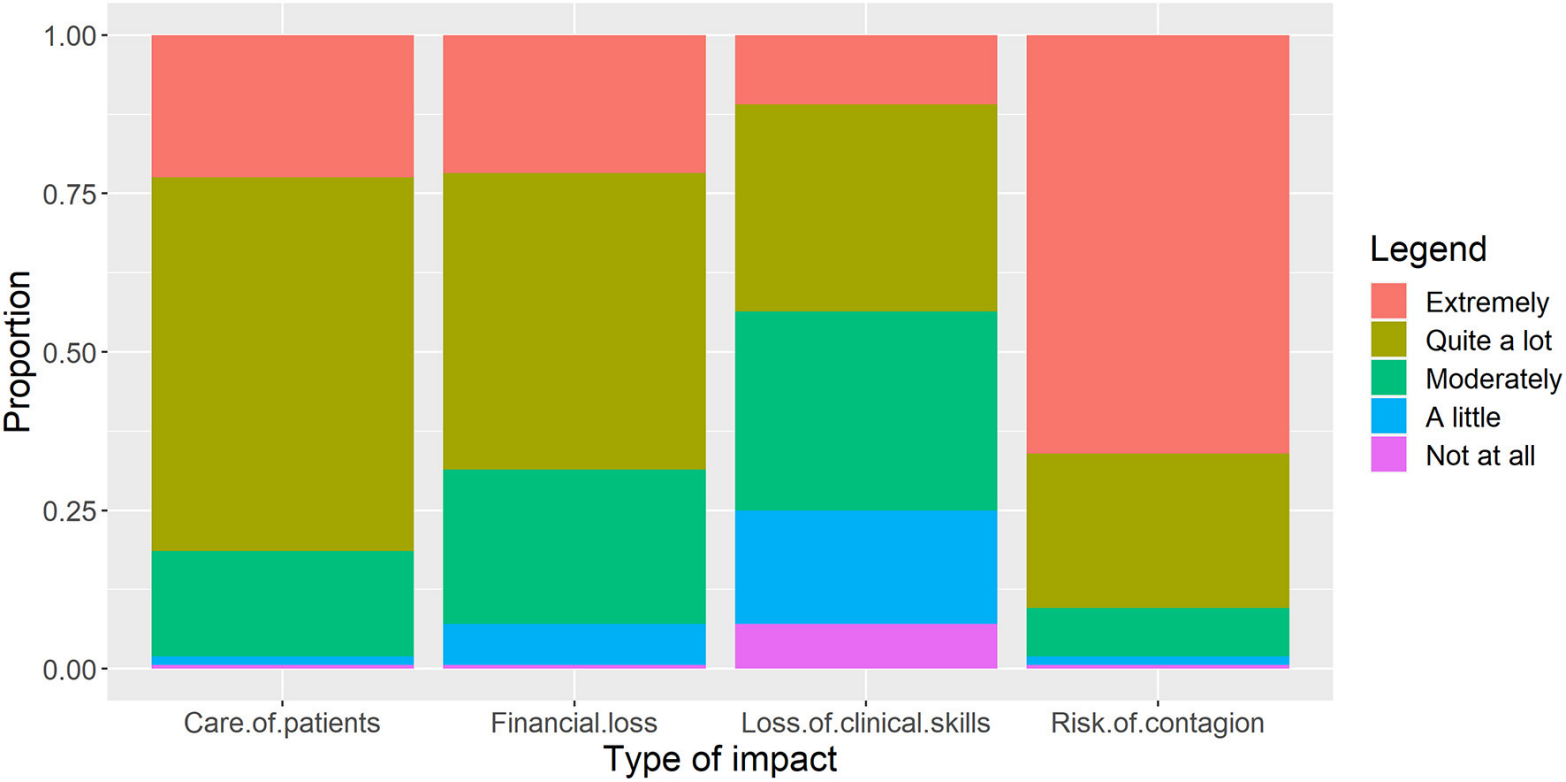
Participant responses about “perceived impacts of COVID-19 on their profession” (*n* = 312). Proportions among respondents are reported.

The results suggest that a large proportion of the respondents (66%) were “Extremely” worried about the risk of contagion during their clinical practices (Figure 1). More than two-third of the respondents recorded their perceived level of impact as “Quite a lot” (59%) and “Extreme” (22.4%) when asked if the COVID-19 pandemic has affected the level of care provided to the patients.

### Perceived Impacts of COVID-19 Pandemic on Dental Practices

**Figure 2:**
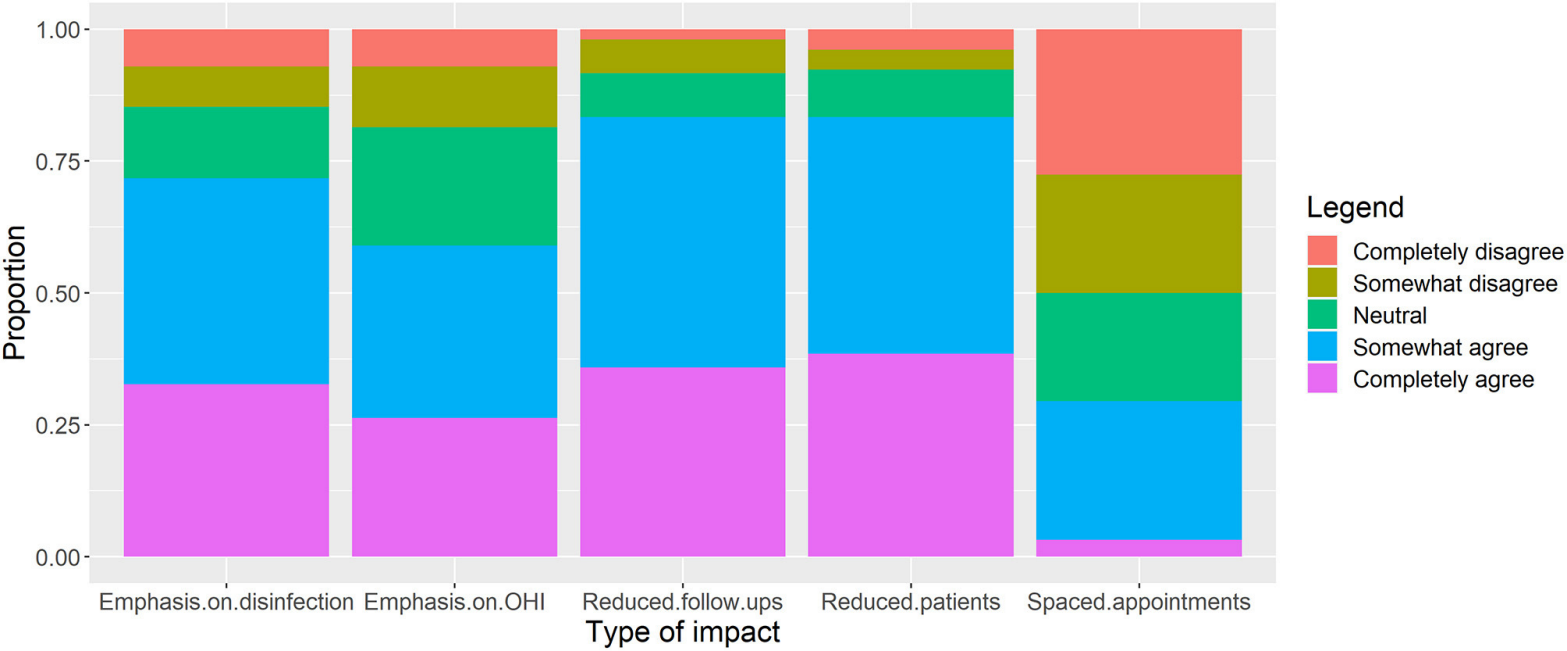
Participant responses about “perceived impacts of COVID-19 on different practices in dentistry” (*n* = 312). Proportions among respondents are reported.

The results suggest that more than two-third of the respondents were either “Completely Agree” or “Somewhat Agree” that the pandemic situation has reduced the number of patients and reduced the number of follow-up visits (Figure 2). Almost two-third of the respondents opined that the pandemic has led to an increased emphasis on the disinfection procedures and OHI in dental practices in Pakistan.

### Perceived Impacts of COVID-19 Pandemic on Procedures in Operative Dentistry

**Figure 3:**
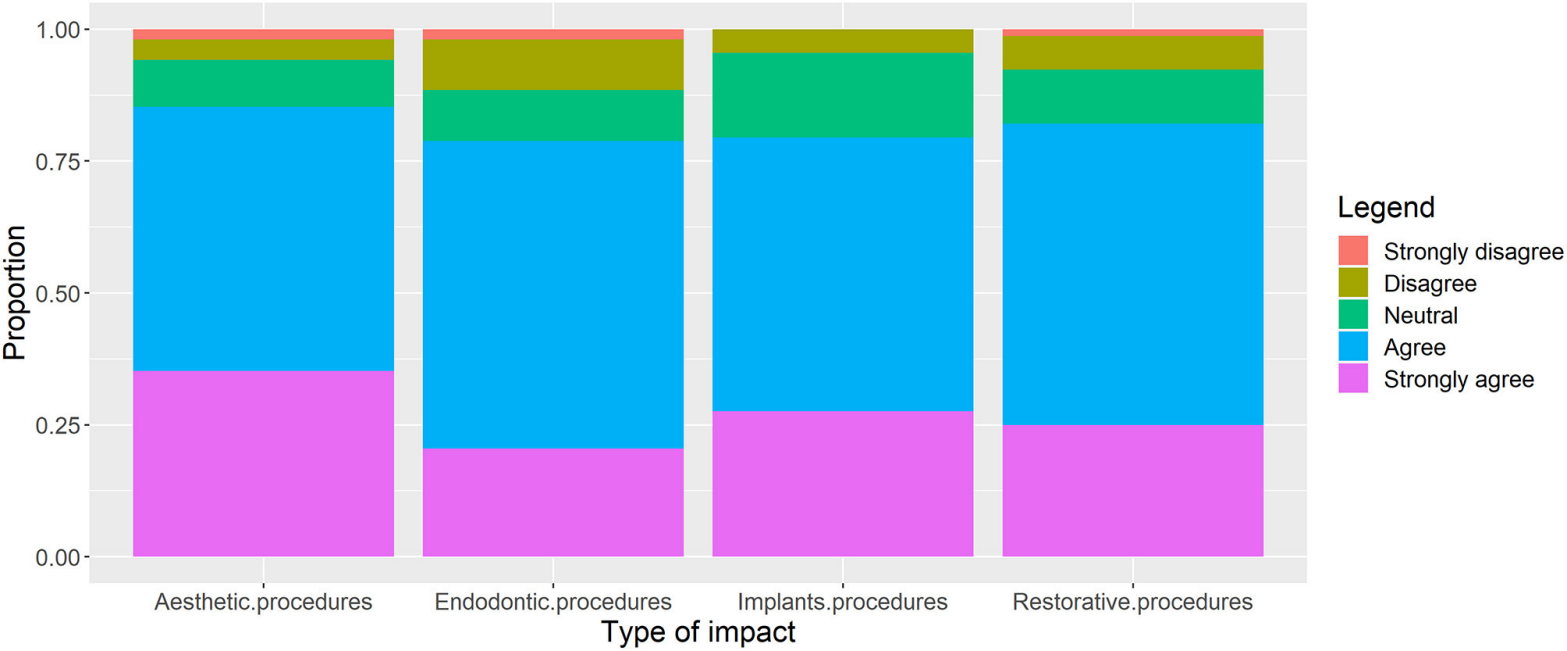
Participant responses about “perceived impacts of COVID-19 on procedures in operative dentistry” (*n* = 312). Proportions among respondents are reported.

More than 75% of the respondents agreed that the pandemic has affected the aesthetic, endodontics, implants and restorative dentistry procedures (Figure 3). The highest agreement was found in response to the question about aesthetic procedures followed by restorative dentistry procedures.

### Regression Modeling (COM-B)

#### Internal Consistency and Computation of Variables

**Figure 4:**
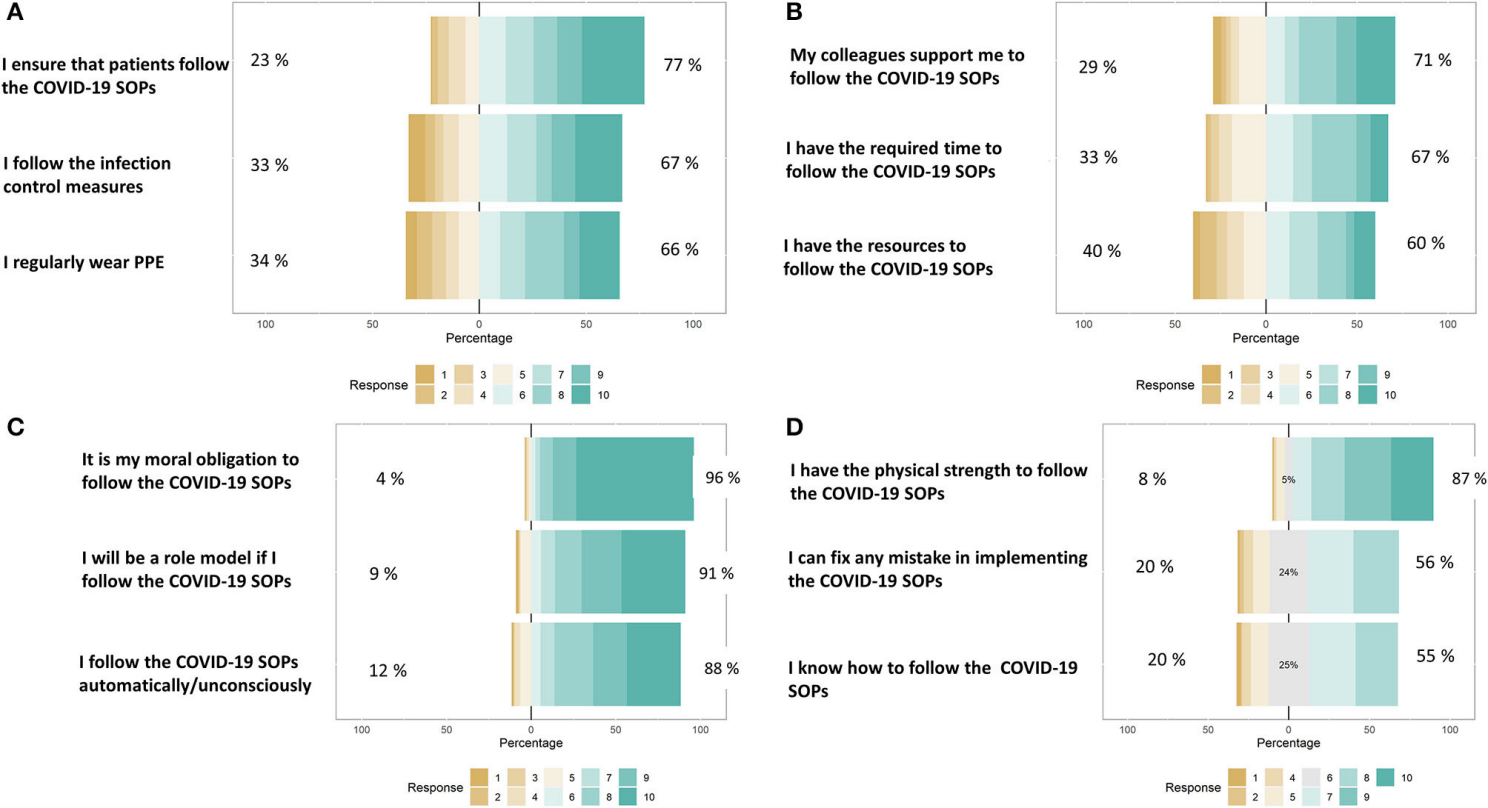
Distribution of responses to the questions asked under the constructs: **(A)** Adherence to COVID-19 SOP's (Behavior), **(B)** Opportunities to follow COVID-19 SOP's, **(C)** Motivation to follow COVID-19 SOP's, and **(D)** Capabilities to follow COVID-19 SOP's. All responses were recorded on a Likert scale (0–10).

Cronbach's alpha is used to assess the internal consistency of the items in each construct (i.e., capability, opportunity, motivation, and behavior). Results suggested an acceptable degree of internal consistency for all four constructs: Capability (0.825), Opportunity (0.801), Motivation (0.707), and Behavior (0.695). Then, “Compute Variable” function in SPSS version 21 (SPSS® (IBM®, SPSS® Statistics, Armonk, NY, USA) was used to compute four continuous variables from the items within each of the four constructs (i.e., the scores of all questions within a construct were averaged and the resulting score was used as a continuous variable). Thus, four continuous variables: Capability, Opportunity, Motivation, and Behavior were generated. Figure 4 shows the response of participants.

#### Multiple Linear Regression Model

Table 3 shows that a significant regression equation was found (*F*_(3, 303)_ = 85.451, *p* < 0.0001), with an adjusted *R*^2^ of 0.453. The regression model shows that the dependent variable is significantly affected by the Opportunities and Capabilities of respondents. With increasing Opportunities, the respondents were significantly more likely to adhere to COVID-19 SOP's (β = 0.494). Similarly, with an increase in the Capabilities of the respondents, there was a significant increase in the Behavior to adhere to COVID-19 SOP's (β = 0.358).

**Table 3:** Multiple regression analysis results showing relationships between dependent variable (Behavior toward adherence to COVID-19 SOPs) and independent variables (Opportunities, Motivation and Capabilities to adhere to COVID-19 SOPs).

**Model**	**Unstandardized**		**Standardized**	***p*-Value**
	**coefficients**		**coefficients**	
	***B***	**Standard Error**	***β***	
(Constant)	−0.977	0.674		0.148
Opportunities	0.516	0.047	0.494	0.000
Motivation	−0.091	0.087	−0.057	0.297
Capabilities	0.612	0.096	0.358	0.000

*Constructs were created from questions in a survey administered to the dentists in Pakistan (n = 312)*.

## Discussion

Healthcare professionals around the world are the most vulnerable groups to COVID-19 exposure as they are most likely to be directly exposed to the infected patients. Healthcare professionals have reported mental stress and physical fatigue due to insufficient health care protection. This study was aimed to understand how the COVID-19 pandemic affected the dentistry professionals in Pakistan, taking a case study of Punjab province in the country. The study also aimed to understand the factors determining the dentists' behavior to adhere to the COVID-19 SOPs.

The results showed that the respondents are most concerned about the risk of contagion. This can be attributed to the fact that the contagious nature of COVID-19 has caused large-scale mortality among the healthcare professionals, especially in the developing countries of South Asia (20). A large number of operative dentistry procedures lead to generation of aerosols due to which dentists are at a high risk of infection (5, 9). Furthermore, scarcity of personal protective equipment also threatens safety of dentists, especially in the developing countries (21). Therefore, it is understandable why most of the respondents in this survey showed highest concern about the risk of contagion.

After the risk of contagion, the respondents were most concerned about inappropriate levels of care provided to the patients. These results are in agreement with earlier reports where dentists in the UK reported a similar concern (5). This could be due to the fact that most of the operative dentistry treatments require a physical presence of the patients (22). However, due to the closure of clinics and dental hospitals, dentists were inaccessible for patients (23). Furthermore, most of the patients, especially those coming from remote rural areas do not have access to the internet for online appointments and follow-ups (24). Thus, it is expected that dentists would show concern about insufficient healthcare provision to their patients.

More than 60% of the respondents also showed high levels of concern about financial loss due to COVID-19 pandemic. The financial aspect of COVID-19 pandemic is not unheard of (25). Several studies have reported that the pandemic situations have caused financial insecurities among health professionals (26, 27). A study in the UK concluded that more than 75% of the dentists are worried about the financial losses caused by the pandemic (5). Similarly, in a large-scale survey of dentists in the Eastern Mediterranean, Europe, North America, and Western Pacific regions, 73.6% of the respondents strongly agreed that the pandemic situations has caused a substantial financial impact on their income (28). In Iraq, a study reported that 75% of dental practitioners believed their income had decreased by as much as 50% due to the pandemic situation (12). In case of developing countries like Pakistan where resources are limited, one could expect financial stress to be a major outcome of the pandemic.

About two-third of the respondents agreed that there has been an increased emphasis on the disinfection procedures and OHI. This could be attributed to the extraordinary awareness campaigns about disinfection procedures (29). Most people, including doctors and the patients, are extremely cautious about the risk of contagion and therefore an unusually high level of emphasis has been made on disinfection procedures in the dental hospitals (30–32). Similar results have been reported in several other studies. In Turkey, for example, dentists reported a significant decrease in the number of patient admitted to dental hospitals (33). The researchers attributed this decrease in the number of patients to the measures taken by dentists and authorities against the COVID-19 pandemic in view of the growing number of cases (33).

In this study, the COM-B model was used to demonstrate how capabilities, opportunities and motivation of dentists predict their COVID-19 adherence behavior. Understanding human behavior in the era of pandemic is critical because behavioral adaptations play a key role in the spread and control of infection (34). Evidence suggests that behavioral science is pivotal to understanding the factors that encourage stakeholders to adopt behaviors that shape the progression of the outbreak (35). The British Psychological Society Behavioral Science and Disease Prevention Taskforce recommends understanding the behavioral adaptations regarding adherence to COVID-19 SOPs through COM-B model of behavior change. Application of the COM-B model to COVID-19 transmission-related behavior will provide a “behavioral diagnosis” that can help us identify the factors most likely to influence the behavior of individuals (13) and, thus, identify appropriate targets for behavior change interventions. The identified behavioral change interventions can then be designed and implemented to improve adherence to preventive behaviors during the period of social isolation.

The results of the regression model showed that the behavior is significantly affected by capabilities and opportunities. Various studies on this subject have reported contrasting results. For example, a study in the UK reported that motivation is the strongest predictor of an individual's behavior to adhere to COVID-19 SOPs (34). In this study, however, motivation was not a significant determinant of behavior. Instead, opportunities and capabilities were the strongest predictors in the regression model. This can be attributed to the fact that in countries with scarce resources, opportunities and capabilities of individuals are often more important than their motivation. Due to lack of resources and feasible environment, people fail to adopt or avoid a behavior despite motivation. The COM-B model gives a theoretical insight of the drivers of COVID-19 SOPs adherence behavior. In the recent past, COM-B model has been extensively used by researchers to model behavior to adhere to different COVID-19 SOPs (5, 34, 36).

This study makes an argument that the policymakers must emphasize more on improving the facilities, infrastructures, and resources for dentists to adopt COVID-19 SOPs. This is because the regression model in the study highlighted the fact that even though the respondents reported high levels of motivation, their behavior was not significantly affected by their motivation. Instead, better capabilities and opportunities lead to a more promising behavior change. Therefore, the scarce financial resources must be spent on providing PPE and other essential equipment and trainings to enable dentists to counter a pandemic situation.

### Limitations of the Study

The study was conducted in the Punjab province of Pakistan. Therefore, the results of this study should not be generalized to other provinces of Pakistan, especially where socioeconomic profile of population is different and where severity of pandemic was different. Furthermore, most of the respondents in this study are under 40 years of age. Under ideal circumstances, a more representative sample of Pakistani dentists in terms of age and years of work experience would have been desirable. However, this study was conducted during periods of lock-down where meeting dentists in-person was not possible. The questionnaires were disseminated through WhatsApp groups and response was completely voluntary. However, the authors believe it did not affect the outcomes as this study did not include any hypothesis regarding the age, gender, or work experience of the dentists. Moreover, most of the dentists above 50 years of age did not attend clinics and hospitals because of being most vulnerable to COVID. Therefore, it is understandable that only younger dentists responded to this questionnaire as they were the ones who practiced dentistry during epidemic and were able to respond to the questions about the impacts of COVID-19 on dentistry profession in Pakistan.

## Conclusion

This study concludes that COVID-19 pandemic has caused considerable worry to operative dentistry professionals in Pakistan. Dentists in the Punjab province of Pakistan reported financial loss, increased focus on disinfections procedures as major outcomes of the pandemic. The study further concludes that there is a need to spend more resources on providing opportunities and improving capabilities of dentists to allow them to successfully follow the COVID-19 guidelines during their clinical practices.

## Data Availability Statement

The raw data supporting the conclusions of this article will be made available by the authors, without undue reservation.

## Ethics Statement

The questionnaire was approved by the Institutional Research Board (IRB) of Bakhtawar Amin Dental College & Hospital Multan, Pakistan (reference BADC&H No. 300/21). The patients/participants provided their written informed consent to participate in this study.

## Author Contributions

SM: writing original draft, research, and analysis. A-HA: analysis, editing, review, and supervision. Both authors contributed to the article and approved the submitted version.

## Conflict of Interest

The authors declare that the research was conducted in the absence of any commercial or financial relationships that could be construed as a potential conflict of interest.

## Publisher's Note

All claims expressed in this article are solely those of the authors and do not necessarily represent those of their affiliated organizations, or those of the publisher, the editors and the reviewers. Any product that may be evaluated in this article, or claim that may be made by its manufacturer, is not guaranteed or endorsed by the publisher.

## References

[1] sars-cov-2-sorveglianza-dati, @ www.epicentro.iss.it [Internet]. Available online at: https://www.epicentro.iss.it/coronavirus/sars-cov-2-sorveglianza-dati (acessed April 20, 2022).

[2] Passarelli PC Rella E Manicone PF Garcia-Godoy F D'Addona A. The impact of the COVID-19 infection in dentistry. Exp Biol Med. (2020) 245:940–4. 10.1177/1535370220928905 32436748 PMC7427177

[3] Spagnuolo G De Vito D Rengo S Tatullo M. COVID-19 outbreak: an overview on dentistry. Int J Environ Res Public Health. (2020) 17:3–5. 10.3390/ijerph17062094 32235685 PMC7143628

[4] Liu Y Ning Z Chen Y Guo M Liu Y Gali NK. Aerodynamic analysis of SARS-CoV-2 in two Wuhan hospitals. Nature. (2020) 582:557–60. 10.1038/s41586-020-2271-3 32340022

[5] Nibali L Ide M Ng D Buontempo Z Clayton Y Asimakopoulou K. The perceived impact of Covid-19 on periodontal practice in the United Kingdom: a questionnaire study. J Dent. (2020) 102:103481. 10.1016/j.jdent.2020.103481 32979456 PMC7510560

[6] Chasib NH Alshami ML Gul SS Abdulbaqi HR Abdulkareem AA Al-Khdairy SA. Dentists' practices and attitudes toward using personal protection equipment and associated drawbacks and cost implications during the COVID-19 pandemic. Front Public Heal. (2021) 9:1–7. 10.3389/fpubh.2021.770164 34869182 PMC8637868

[7] Bescos R Casas-Agustench P Belfield L Brookes Z Gabaldón T. Coronavirus disease 2019 (COVID-19): emerging and future challenges for dental and oral medicine. J Dent Res. (2020) 99:1113. 10.1177/0022034520932149 32463715

[8] Veena HR Mahantesha S Joseph PA Patil SR Patil SH. Dissemination of aerosol and splatter during ultrasonic scaling: a pilot study. J Infect Public Health. (2015) 8:260–5. 10.1016/j.jiph.2014.11.004 25564419

[9] Madathil S Siqueira WL Marin LM Sanaulla FB Faraj N Quiñonez CR. The incidence of COVID-19 among dentists practicing in the community in Canada: a prospective cohort study over a six-month period. J Am Dent Assoc. (2021) 153:450–9.e1. 10.1016/j.adaj.2021.10.006 35241268 PMC8565357

[10] Das G Samejo I Ahmed S Jabeen B Shaikh MI Mushtaque K. Cross infection control in private dental practice in Karachi, Sindh. Prof Med J. (2019) 26:1354–8. 10.29309/TPMJ/2019.26.08.17

[11] NHS England NHS Improvement. COVID-19 guidance and standard operating procedure urgent dental care systems in the context of coronavirus. Delay Phase NHS Engl. (2020) 2:2–35. Available online at: https://go.digitalsmiledesign.com/hubfs/BIOSAFETY/C0282-covid-19-urgent-dental-care-sop.pdf (accessed September 30, 2021).

[12] Mahdee AF Gul SS Abdulkareem AA Qasim SSB. Anxiety, practice modification, and economic impact among iraqi dentists during the COVID-19 outbreak. Front Med. (2020) 7:595028. 10.3389/fmed.2020.595028 33425944 PMC7793761

[13] Michie S Van Stralen MM West R. The behaviour change wheel: a new method for characterising and designing behaviour change interventions. Implement Sci. (2011) 6:1–12. 10.1186/1748-5908-6-42 21513547 PMC3096582

[14] Asimakopoulou K Newton JT. The contributions of behaviour change science towards dental public health practice: a new paradigm. Community Dent Oral Epidemiol. (2015) 43:2–8. 10.1111/cdoe.12131 25327392

[15] Loch C Kuan IBJ Elsalem L Schwass D Brunton PA Jum'ah A. COVID-19 and dental clinical practice: students and clinical staff perceptions of health risks and educational impact. J Dent Educ. (2021) 85:44–52. 10.1002/jdd.12402 32914437

[16] Al-Khalifa KS AlSheikh R Al-Swuailem AS Alkhalifa MS Al-Johani MH Al-Moumen SA. Pandemic preparedness of dentists against coronavirus disease: a Saudi Arabian experience. PLoS ONE. (2020) 15:e0237630. 10.1371/journal.pone.0237630 32813692 PMC7437908

[17] Kamran R Saba K Azam S. Impact of COVID-19 on Pakistani dentists: a nationwide cross sectional study. BMC Oral Health. (2021) 21:59. 10.1186/s12903-021-01413-6 33568128 PMC7874993

[18] Qamar MK Shaikh BT Afzal A. What do the dental students know about infection control? A cross-sectional study in a teaching hospital, Rawalpindi, Pakistan. Biomed Res Int. (2020) 2020:3413087. 10.1155/2020/3413087 32596299 PMC7285392

[19] Yamane T. Statistics, An Introductory Analysis. 2nd ed. New York, NY: Harper and Row (1967).

[20] Jatoi NN Ahmad S Sajid E ud din Yasmin F Asghar MS Farhan SA. Are doctors protected enough during COVID-19 in South Asia? Glob Heal Res Policy. (2021) 6:36. 10.1186/s41256-021-00219-x 34593053 PMC8481110

[21] Spinazzè A Cattaneo A Cavallo DM. COVID-19 outbreak in Italy: protecting worker health and the response of the Italian Industrial Hygienists Association. Ann Work Expo Health. (2020) 64:559–64. 10.1093/annweh/wxaa044 32298415 PMC7184418

[22] Gambarini E Galli M Di Nardo D Miccoli G Patil S Bhandi S. A survey on perceived COVID-19 risk in dentistry and the possible use of rapid tests. J Contemp Dent Pract. (2020) 21:718–22. 10.5005/jp-journals-10024-2851 33020352

[23] Welti R D'Mello G Ramalingam L Silva M. COVID-19: implications for paediatric dental care in the hospital setting. J Paediatr Child Health. (2020) 56:1661–2. 10.1111/jpc.15198 32959944 PMC7536949

[24] Nuvvula S Mallineni SK. Remote management of dental problems in children during and post the COVID-19 pandemic outbreak: a teledentistry approach. Dent Med Probl. (2021) 58:237–41. 10.17219/dmp/133182 34019745

[25] Gasparro R Scandurra C Maldonato NM Dolce P Bochicchio V Valletta A. Perceived job insecurity and depressive symptoms among Italian dentists: the moderating role of fear of COVID-19. Int J Environ Res Public Health. (2020) 17:5338. 10.3390/ijerph17155338 32722202 PMC7432196

[26] Amato A Ciacci C Martina S Caggiano M Amato M. COVID-19: the dentists' perceived impact on the dental practice. Eur J Dent. (2021) 15:469–74. 10.1055/s-0041-1734470 33622007 PMC8413015

[27] Ahmadi H Ebrahimi A Ghorbani F. The impact of COVID-19 pandemic on dental practice in Iran: a questionnaire-based report. BMC Oral Health. (2020) 20:1–9. 10.1186/s12903-020-01341-x 33272261 PMC7711254

[28] Bakaeen LG Masri R AlTarawneh S Garcia LT AlHadidi A Khamis AH. Dentists' knowledge, attitudes, and professional behavior toward the COVID-19 pandemic: a multisite survey of dentists' perspectives. J Am Dent Assoc. (2021) 152:16–24. 10.1016/j.adaj.2020.09.022 33250171 PMC7524642

[29] Alzyood M Jackson D Aveyard H Brooke J. COVID-19 reinforces the importance of handwashing. J Clin Nurs. (2020) 29:2760–1. 10.1111/jocn.15313 32406958 PMC7267118

[30] Israel S Harpaz K Radvogin E Schwartz C Gross I Mazeh H. Dramatically improved hand hygiene performance rates at time of coronavirus pandemic. Clin Microbiol Infect. (2020) 26:1566. 10.1016/j.cmi.2020.06.002 32526277 PMC7831641

[31] Li G Chang B Li H Wang R Li G. Precautions in dentistry against the outbreak of corona virus disease 2019. J Infect Public Health. (2020) 13:1805–10. 10.1016/j.jiph.2020.09.013 33069661 PMC7539802

[32] Alomari AH Aga O El Sahmarany L Hegazi M Almulla L. Public perception towards medical waste generated in the environment during the COVID-19 pandemic in Eastern Province, Saudi Arabia. Heliyon. (2021) 7:e08363. 10.1016/j.heliyon.2021.e08363 34786514 PMC8580859

[33] Duruk G Gümüşboga ZS Çolak C. Investigation of Turkish dentists' clinical attitudes and behaviors towards the COVID-19 pandemic: a survey study. Braz Oral Res. (2020) 34:e054. 10.1590/1807-3107bor-2020.vol34.0054 32490887

[34] Gibson Miller J Hartman TK Levita L Martinez AP Mason L McBride O. Capability, opportunity, and motivation to enact hygienic practices in the early stages of the COVID-19 outbreak in the United Kingdom. Br J Health Psychol. (2020) 25:856–64. 10.1111/bjhp.12426 32415918 PMC7276910

[35] West R Michie S Rubin GJ Amlôt R. Applying principles of behaviour change to reduce SARS-CoV-2 transmission. Nat Hum Behav. (2020) 4:451–9. 10.1038/s41562-020-0887-9 32377018

[36] Schmidtke KA Drinkwater Drinkwater KG A cross-sectional survey assessing the influence of theoretically informed behavioural factors on hand hygiene across seven countries during the COVID-19 pandemic. BMC Public Health. (2021) 21:1–14. 10.1186/s12889-021-11491-4 34289816 PMC8293513

